# Supplementary material for: A web-database of mammalian morphology and a reanalysis of placental phylogeny
Source: BMC Evol Biol. 2007 Jul 3;7:108. doi: 10.1186/1471-2148-7-108 (PMC1941728; doi:10.1186/1471-2148-7-108)
Supplement: Additional File 1 — Website data: The 196 characters first described in [17], along with the supplementary data indicated below, have been joined into a number of HTML files, all of which are linked to the "morphsite_bmc07.html" index page. The embedded links to the supporting files are functional, independent of PC or platform, provided that the directory structure remains intact (i.e., all files remain in a single folder, no subdirectories). Nexus files: Datafiles containing the morphological, indel, and aligned DNA sequence data, linked from the "morphsite_bmc07.html" index page. Table 1. Changes to the multiple sequence alignment of [9]. Table 2. Qualitative character-state corrections to the morphology matrix of [17]. Table 3. Summary of discrepancies between Appendices 1 and 2 of [17]. [file 1471-2148-7-108-S1.zip › asher-BMC07-website/softtissues.html]

asher-characterBank


|  |  |  |
| --- | --- | --- |
| Soft Tissue |  | This work is licensed under a   Creative Commons License. |
| Soft tissue  186 forelimb patagium  ;  0 absent 1 present,   187 cloaca ;  0 anal-urogenital openings separate 1 present,   188 trophoblast  ;  0 abs 1 pres,   189 maternal-fetal nutrient exchange;  0 choriovitelline 1 chorioallantoic,   190 placental membrane ;  0 epitheliochorial 1 endotheliochorial 2 haemochorial,   191 yolk sac ;  0 permanent 1 temporary 2 rudimentary,   192 gestation time  ;  0 short 1 prolonged,   193 ureter-muellerian ducts  ;  0 medial 1 lateral,   194 hindgut  ;  0 differentiated 1 simplified,   195 testes ;  0 abdominal 1 descend-ascrotal 2 descend-scrotal,   196 eustacian sac  ;  0 abs 1 present |  |  |
